# Alternative nucleic acid structures in microbial biology: regulatory functions and antimicrobial opportunities

**DOI:** 10.3389/fcimb.2026.1852766

**Published:** 2026-07-29

**Authors:** Sabreena Yousuf, Misba M., Saleem Bhat

**Affiliations:** 1 University of Kashmir, Srinagar, India; 2 University of Pennsylvania, Philadelphia, PA, United States

**Keywords:** alternative DNA structures, antibiotic resistance, antimicrobial targets, G-quadruplex, microbial gene regulation, R-loops, RNA structure

## Abstract

The rapid emergence and global dissemination of antimicrobial resistance (AMR) pose a critical threat to public health, necessitating the identification of unconventional biological targets and regulatory mechanisms in microbial pathogens. Beyond the canonical Watson–Crick double helix, nucleic acids can adopt a wide range of alternative DNA and RNA structures, including G-quadruplexes, Z-DNA, cruciforms, triplex DNA, R-loops, and selected RNA structures, including RNA G-quadruplexes and RNA–DNA hybrids. These structures are increasingly recognized as dynamic and functional elements in microbial genomes and transcriptomes, where they influence gene regulation, genome stability, stress adaptation, and pathogenicity. Importantly, many of these processes are directly linked to antibiotic tolerance, persistence, and resistance evolution. This review provides a comprehensive and critical overview of alternative nucleic acid structures in bacteria, archaea, and microbial eukaryotes, with a particular emphasis on their relevance to antibiotic resistance and antimicrobial drug development. We summarize updated knowledge of their formation, biological roles, and regulatory mechanisms, discuss emerging methodologies for their detection *in vivo*, and highlight their potential as novel antimicrobial targets. By integrating structural nucleic acid biology with infection microbiology, this review aims to frame non-canonical nucleic acid architectures as an underexploited layer of microbial regulation with significant translational potential.

## 1 Introduction

Antimicrobial resistance (AMR) represents one of the most serious global health challenges of the twenty-first century, threatening the effective prevention and treatment of a wide range of infectious diseases (Murray et al., 2019; Jia et al., 2025). The relentless rise of multidrug-resistant bacteria has eroded the efficacy of nearly all major antibiotic classes, while the pace of discovery of new antimicrobial agents has slowed dramatically. Traditional antibiotic development has focused primarily on a limited set of protein targets involved in essential cellular processes, such as cell wall biosynthesis, protein translation, DNA replication, and core metabolic pathways (Tipper and Strominger, 1965; Shen and Pernet, 1985; Shen et al., 1989; Moazed and Noller, 1987; Gladki et al., 2013; Jia et al., 2025). Although these targets have historically yielded clinically successful drugs, their repeated exploitation has driven the rapid evolution and dissemination of resistance mechanisms (Toprak et al., 2012).

In response to this crisis, there is growing recognition that fundamentally new conceptual approaches are required to identify antimicrobial targets and strategies that are less prone to resistance development (Stokes et al., 2020). One promising direction is the exploration of regulatory layers that control microbial adaptability, stress responses, and evolutionary plasticity, rather than targeting viability alone (Stokes et al., 2020; Masi et al., 2017; MacLean et al., 2013). In this context, nucleic acids are no longer seen only as carriers of genetic information but also as dynamic molecules that can adopt different structures with distinct biological function (Rawal et al., 2006; Sultan et al., 2025).

**Figure 1:**
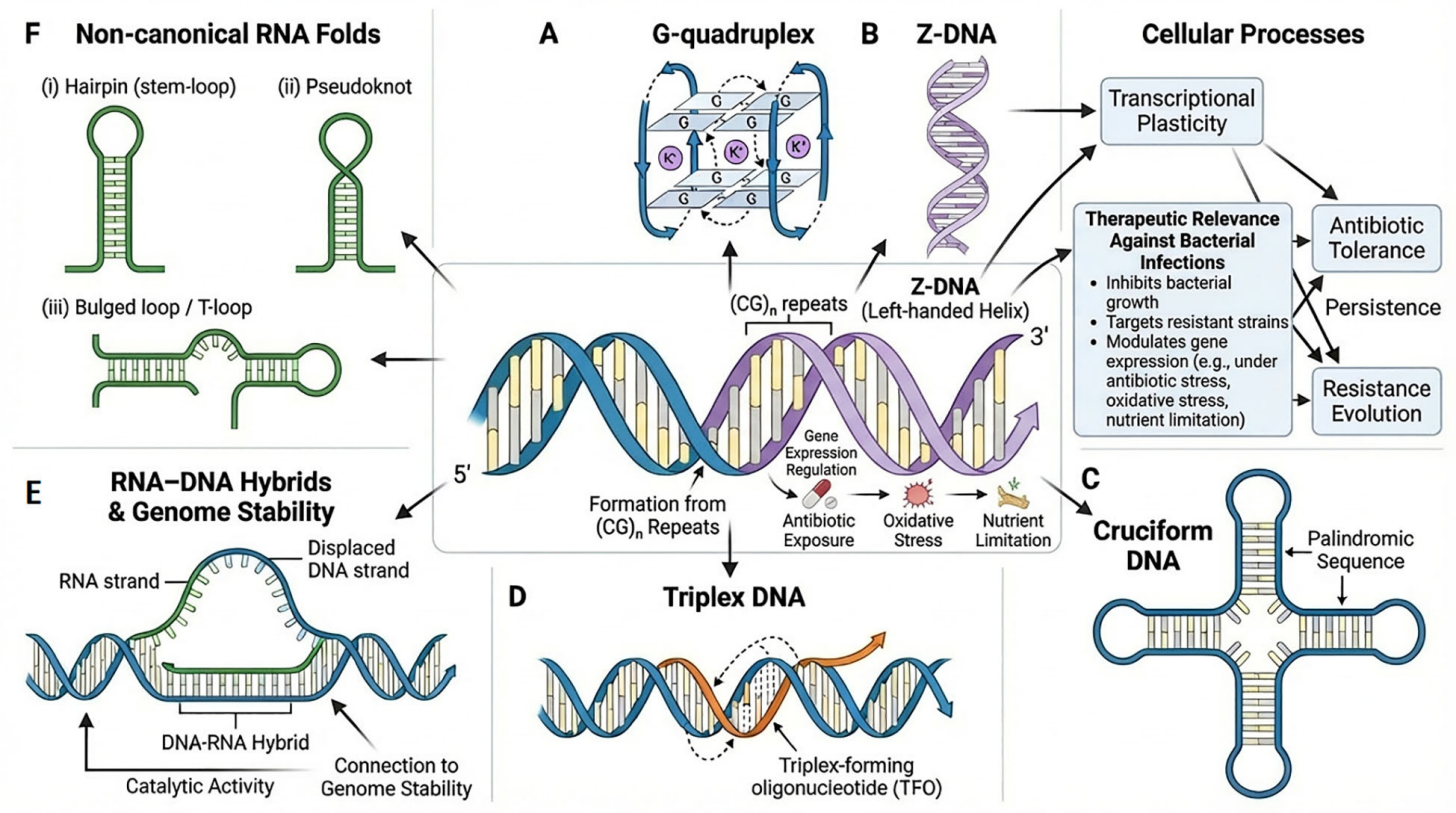
Alternative nucleic acid structures in bacterial gene regulation and stress responses. **(A–D)** Non-canonical DNA structures: **(A)** G-quadruplex formed from G-rich sequences and stabilized by K^+^; **(B)** Z-DNA arising in GC-rich, negatively supercoiled regions; **(C)** Cruciform DNA formed at palindromic sequences; and **(D)** Triplex DNA generated by third-strand binding (TFOs). **(E)** R-loop showing an RNA–DNA hybrid with displaced ssDNA, linked to genome stability. **(F)** Non-canonical RNA folds including hairpins, pseudoknots, and bulged loops. Structure formation from (CG)_n_ repeats and stress conditions (antibiotics, oxidative stress, nutrient limitation) modulates gene expression, contributing to transcriptional plasticity, antibiotic tolerance, persistence, and resistance evolution.

Beyond the canonical Watson–Crick B-form DNA double helix and simple RNA secondary structures, nucleic acids can form a diverse repertoire of non-canonical architectures, including G-quadruplexes, Z-DNA, cruciforms, triplex DNA, R-loops, riboswitches, pseudoknots, and RNA G-quadruplexes (Drolet et al., 1994; Krishna Leela et al., 2013; Hänsel-Hertsch et al., 2016; Raghunathan et al., 2018; Shao et al., 2020; Kumar et al., 2021). These alternative nucleic acid structures (**Figure 1**) are not merely rare or pathological intermediates; rather, they arise naturally under physiological conditions driven by sequence composition, DNA supercoiling, transcriptional activity, ionic environment, and interactions with proteins and small molecules (Rawal et al., 2006; Kouzine and Levens, 2007; Howe et al., 2015; Hänsel-Hertsch et al., 2016). In microbial genomes, which are compact, densely transcribed, and often under strong selective pressure, such structures can form frequently and exert significant regulatory effects (Raghunathan et al., 2018; Shao et al., 2020).

Importantly, many cellular processes influenced by alternative nucleic acid structures such as transcriptional plasticity, genome stability, stress-induced mutagenesis, horizontal gene transfer, and phenotypic heterogeneity are directly linked to antibiotic tolerance, persistence, and resistance evolution (**Figure 1**) (Lombardo et al., 2004; Ponder et al., 2005; Palmer and Kishony, 2013; Rodríguez-Rojas et al., 2014). For example, transcriptional reprogramming under antibiotic stress can promote the formation of non-B DNA structures and R-loops, which in turn influence mutation rates and genome rearrangements (Ponder et al., 2005; MacLean et al., 2013; Bacolla et al., 2016; Lang et al., 2017). Similarly, structured regulatory RNAs can rapidly modulate the expression of resistance determinants without requiring new protein synthesis (Roth et al., 2014; Tian et al., 2023). Recent evidence from multiple studies demonstrate RNA secondary structures modulate translation termination and stop codon readthrough (Ghelfi et al., 2023; Bhat et al., 2025; Kansara et al., 2026).

Despite accumulating evidence for their functional relevance, alternative nucleic acid structures remain underrepresented in antimicrobial research and drug development pipelines (Krishna Leela et al., 2013; Shao et al., 2020). Compared with protein targets, they present distinct challenges but also unique opportunities: they often regulate multiple downstream pathways, can be targeted by small molecules in a structure-specific manner, and may impose a higher fitness cost when disrupted, potentially limiting resistance emergence (Rodríguez-Rojas et al., 2014; Sultan et al., 2025).

This review provides an in-depth and critical analysis of alternative nucleic acid structures in microbial biology, with a particular emphasis on their relevance to antibiotic resistance and the development of new antimicrobial strategies (Gifford et al., 2018). We expand upon the molecular basis of structure formation, summarize evidence linking specific non-canonical DNA and RNA structures to resistance-associated processes, and discuss emerging therapeutic and biotechnological opportunities. By integrating insights from structural biology, microbiology, and infection research, we aim to position alternative nucleic acid architectures as a central, yet underexploited, layer of microbial regulation with significant implications for combating antimicrobial resistance.

## 2 Structural plasticity of nucleic acids in microbial systems

Nucleic acids are not static informational polymers but highly dynamic macromolecules capable of adopting diverse conformations in response to sequence context and cellular conditions (Rawal et al., 2006; Wells, 2009; Shao et al., 2020; Kumar et al., 2021). In microbial cells, the chromosome exists in a compacted yet topologically active state shaped by continuous transcription, replication, and environmental fluctuation (Lang et al., 2017; Xu et al., 2021). This dynamic environment lowers the energetic threshold for deviations from canonical B-form DNA and promotes the formation of alternative secondary and tertiary structures (Sudarsan et al., 2008; Nelson et al., 2013; Monsen et al., 2022; Sultan et al., 2025). Structural plasticity, therefore, represents an intrinsic property of microbial genomes rather than a rare exception (Pyne et al., 2021; Janissen et al., 2024).

### 2.1 Sequence determinants of alternative structure formation

Intrinsic sequence composition is a primary driver of structural diversification. Guanine-rich regions predispose loci to G-quadruplex formation, while inverted repeats facilitate cruciform extrusion through intra-strand base pairing (Laughlan et al., 1994; Herbert and Rich, 1996; Shlyakhtenko et al., 1998; Garg et al., 2016). Alternating purine–pyrimidine tracts favor transitions to left-handed Z-DNA (**Figure 1**), and homopurine–homopyrimidine stretches can support triplex (H-DNA) formation under superhelical stress (Lilley, 1980; Stirdivant et al., 1982; Mirkin and Frank-Kamenetskii, 1994; Huppert and Balasubramanian, 2007). Additionally, GC-rich or repetitive regions are particularly prone to strand separation during active transcription, increasing the likelihood of RNA–DNA hybrid formation and R-loop stabilization (Duprey and Groisman, 2020; Pyne et al., 2021; Janissen et al., 2024).

Importantly, these motifs are not randomly distributed across bacterial chromosomes. Bioinformatic analyses reveal enrichment near promoters, regulatory regions, replication origins, terminators, and mobile genetic elements. Such localization suggests that structural potential has been evolutionarily retained in regions where regulatory flexibility or genome plasticity confers adaptive benefit (Mirkin and Frank-Kamenetskii, 1994). In this sense, sequence architecture therefore encodes both genetic information and latent conformational potential that can be selectively deployed under physiological stress (Pyne et al., 2021; Janissen et al., 2024). Of special interest is the recent demonstration of RNA transcripts regulating G-quadruplex landscapes through G-loop formation (Sato et al., 2025). Structural plasticity therefore represents an intrinsic thermodynamic and regulatory property of microbial genomes rather than a rare structural exception.

### 2.2 Topological and environmental drivers

DNA topology plays an equally critical role in shaping structural outcomes. Negative supercoiling, introduced primarily by DNA gyrase and modulated by topoisomerases, reduces the energetic barrier required for local strand separation and conformational transitions (Dai et al., 1998; Kouzine and Levens, 2007; Liang et al., 2024). Rapid growth, high transcriptional output, and replication–transcription collisions generate torsional stress that can trigger transient formation of noncanonical structures (Tsao et al., 1989; Meyer and Beslon, 2014; Janissen et al., 2024).

Environmental conditions further influence these dynamics. Changes in ionic strength, oxidative stress, nutrient limitation, and antibiotic exposure alter transcriptional programs and supercoiling levels, indirectly promoting structural rearrangements (Gifford et al., 2018). For example, antibiotics that inhibit DNA gyrase, RNA polymerase, or ribosomal function can disrupt topological homeostasis (Shen, 1994; Lee et al., 2025) and increase the probability of alternative structure formation (Merrikh et al., 2011; Duprey and Groisman, 2020). Thus, structural plasticity is intimately linked to cellular stress responses.

### 2.3 RNA conformational diversity

RNA molecules exhibit even greater conformational diversity due to their single-stranded nature and extensive capacity for intra- and intermolecular base pairing. In bacteria, RNA folding occurs co-transcriptionally and is tightly coupled to translation and RNA decay. Structured RNAs can function as riboswitches, thermosensors, scaffolds, and catalytic ribozymes, enabling rapid regulatory responses without requiring additional protein factors. RNA–DNA hybrids further expand this structural repertoire, connecting transcriptional dynamics to genome stability (Watters et al., 2016; Winkler et al., 2002). Collectively, these features underscore that alternative nucleic acid conformations are intrinsic and pervasive components of microbial genomes and transcriptomes. Their formation reflects a finely tuned balance between regulatory utility and the imperative to maintain genome integrity. This equilibrium becomes particularly significant under antibiotic stress and during resistance evolution (Lang et al., 2017; Chacón et al., 2026).

## 3 Alternative DNA structures in microbial genomes and their functional roles

### 3.1 G-Quadruplex DNA: distribution, regulation, and functional impact

G-quadruplexes (G4s) are non-canonical four-stranded nucleic acid structures formed by stacked guanine tetrads stabilized by Hoogsteen hydrogen bonding and monovalent cations (K^+^/Na^+^) (Rawal et al., 2006). Once thought to be limited to telomeric DNA, G4s are now recognized as evolutionarily conserved elements widely distributed across bacterial, archaeal, and eukaryotic genomes, with enrichment in regulatory regions such as promoters, untranslated regions (UTRs), and replication origins (Wu et al., 2021; Lyu et al., 2022).

In microbes, G4s regulate gene expression at multiple levels. DNA G4s in promoter regions can modulate transcription by altering DNA topology, restricting transcription factor binding, or impeding RNA polymerase (Biswas et al., 2016; Sultan et al., 2025). RNA G4s within transcripts further influence translation efficiency and mRNA stability. These structures are dynamic and responsive to environmental cues, such as ionic changes, oxidative stress, and nutrient availability. This allows G4s to function as conditional regulatory switches (Biswas et al., 2016; Shao et al., 2020; Wu et al., 2021).

G4s are also implicated in pathways relevant to antibiotic resistance and persistence, including biofilm formation, quorum sensing, and multidrug efflux. Modulating G4 stability in these regions can disrupt gene expression programs required for stress adaptation, potentially sensitizing bacteria to antibiotics (Biswas et al., 2016; Shao et al., 2020; Wu et al., 2021). Because this approach targets nucleic acid structure rather than proteins, it may bypass conventional resistance mechanisms. Pertinently, recent evidence indicates that G-quadruplex structures can directly modulate RNA regulation, linking bacterial G4 biology to post-transcriptional control and potential antimicrobial targeting (Beaume et al., 2013; Singh et al., 2025).

Therapeutically, G4s are established targets in medicine, where small molecules stabilize these structures via π–π stacking and groove interactions (Cadoni et al., 2021; Long et al., 2022; Sultan et al., 2025). Extending this strategy to antimicrobials is promising but requires overcoming challenges such as selectivity over host G4s, cellular permeability, and efflux. Structural differences between microbial and human G4s may enable rational ligand design (Siddiqui-Jain et al., 2002; Biswas et al., 2016; Shao et al., 2020; Wu et al., 2021). Recent structure-based studies have demonstrated that G-quadruplexes in bacteria can be selectively targeted by small molecules, highlighting the therapeutic potential of bacterial G4-directed antimicrobial strategies (Fiabane et al., 2025). Overall, targeting G-quadruplexes represents a shift from protein inhibition to modulation of genome architecture and regulatory networks, offering a potential avenue for next-generation antimicrobial development (Beaume et al., 2013).

### 3.2 Z-DNA and transcription-driven structural transitions

Z-DNA is a left-handed DNA conformation characterized by a zigzag phosphate backbone and altered groove geometry relative to canonical B-DNA. It forms preferentially at alternating purine–pyrimidine sequences, especially (CG)n tracts, under conditions of negative supercoiling or elevated ionic strength (Hamada et al., 1982; Nordheim et al., 1982; Wang and Hogan, 1985). In bacteria, transcription and replication generate localized torsional stress, creating transient topological environments that can promote B-to-Z transitions (Wu et al., 1988; Liu and Wang, 1987; Kladde et al., 1994; Lukomski and Wells, 1994).

Bioinformatic analyses have identified Z-DNA-forming motifs enriched near promoters, transcription start sites, and recombination-prone regions in diverse bacterial genomes (Kouzine et al., 2017; Beknazarov et al., 2020; Beknazarov and Poptsova, 2023; Makus et al., 2025). This distribution suggests that Z-DNA may serve as a structural modulator of gene expression and DNA topology due to their stability in vivo (Rahmouni and Wells, 1989). One leading hypothesis is that Z-DNA functions as a torsional buffer, absorbing excess negative supercoiling generated during intense transcription. The resulting changes in DNA geometry may also influence RNA polymerase progression and the binding of nucleoid-associated proteins or topoisomerases. Z-DNA is increasingly being implicated to play such roles for viral genome and transcription regulation as well (Romero et al., 2024; Run et al., 2025). In yeast, Z-DNA is seen as a nucleosome-boundary element (Wong et al., 2007). Despite these intriguing observations, direct *in vivo* evidence for a regulatory role of Z-DNA in bacteria remains limited, and most support derives from computational analyses and biochemical studies. Nevertheless, the use of new methods such as, ‘Z-DNA hunter tool’ in fruit fly may emerge as promising strategies for real-time detection of Z-DNA across living systems (Petrovič et al., 2025). Notably, Z-DNA formation in conserved mammalian promoters are associated with increased transcription reinitiation rates (Beknazarov et al., 2024). And Z-DNA-binding proteins are reported to act as strong effectors of gene expression in vivo (Oh et al., 2002). The evolutionary conservation of Z-DNA-prone sequences suggests that their structural potential may contribute to transcriptional regulation, recombination, and stress adaptation. For instance, ZBP1 subcellular localization and association with stress granules is modulated and controlled by its Z-DNA binding domains (Deigendesch et al., 2006). The structural view of B-to-Z DNA transition has begun to emanate displaying that Z-DNA is not just an *in vitro* curiosity but a biologically relevant DNA state involved in transcription, immune signaling, and genome stress responses (de Rosa et al., 2013; Beknazarov et al., 2024; Lee et al., 2026).

From a therapeutic perspective, small molecules that alter B–Z transitions could perturb DNA topology and disrupt coordinated stress-response programs. Although this concept remains largely unexplored, Z-DNA represents a potentially tractable structural target for antimicrobial development.

## 4 R-Loops as drivers of genome plasticity, stress responses, and resistance evolution

R-loops are three-stranded nucleic acid structures composed of an RNA–DNA hybrid and a displaced single-stranded DNA (ssDNA) strand (Jalan et al., 2024; Kuznetsova et al., 2024). They arise when nascent RNA reanneals to its DNA template behind the transcription complex, displacing the non-template strand. Once considered rare transcriptional byproducts, R-loops are now recognized as dynamic intermediates that influence chromosome organization, transcriptional regulation, DNA repair, and genome stability across all domains of life (Lang et al., 2017; Lin et al., 2022; Kuznetsova et al., 2024). In microbial systems, their formation is closely linked to transcription–replication conflicts, elevated transcriptional output, negative DNA supercoiling, and defects in RNA processing or translation–transcription coupling (Merrikh et al., 2012). Importantly, under conditions of antibiotic stress, these triggers are amplified, positioning R-loops at the intersection of stress adaptation and resistance evolution (Lang et al., 2017; Kuznetsova et al., 2024; Chacón et al., 2026). Recent work demonstrated that stress-induced genomic R-loops can promote biofilm extracellular matrix formation by contributing extracellular nucleic acid components following bacterial cell lysis, thereby linking R-loop biology to microbial community adaptation and persistence (Mugunthan et al., 2025).

### 4.1 Mechanisms of R-loop formation in bacteria

In bacteria, R-loops preferentially form at GC-rich sequences, repetitive regions, and highly transcribed operons where the thermodynamic stability of RNA–DNA hybrids is enhanced (**Figure 1**). Strong negative supercoiling behind RNA polymerase further promotes hybrid formation by destabilizing the DNA duplex, facilitating invasion of the nascent RNA strand (Lang et al., 2017; Kuznetsova et al., 2024). During rapid growth, intense transcriptional activity and concurrent DNA replication increase the likelihood of collisions between replication forks and transcription complexes. Such transcription–replication conflicts create favorable topological and structural environments for R-loop initiation (Wimberly et al., 2013; Lang et al., 2017). Recent development of enDR3, an engineered RNase H3 hybrid-binding domain enables sensitive and specific genome-wide R-loop mapping *in vivo* and ex vivo, improving the resolution and reliability of R-loop profiling approaches (Jedynak-Slyvka et al., 2025).

Antibiotic exposure intensifies these processes. Fluoroquinolones interfere with DNA gyrase and topoisomerase IV, altering supercoiling dynamics and increasing replication fork stalling. Rifamycins inhibit transcription elongation, leading to polymerase pausing and accumulation of RNA transcripts near promoters (Ojkic et al., 2020; Lang and Merrikh, 2021; Jia et al., 2025). Aminoglycosides disrupt translation, uncoupling it from transcription and exposing nascent RNA that would otherwise be shielded by ribosomes. Together, these perturbations enhance the probability of RNA reannealing to DNA, thereby increasing R-loop formation under drug pressure (Kouzminova et al., 2017; Lang and Merrikh, 2021).

Bacterial cells tightly regulate R-loop abundance to maintain genomic integrity. RNase H enzymes, particularly RNase HII and RNase HIII in many species, degrade the RNA component of RNA–DNA hybrids, preventing excessive accumulation. Helicases such as UvrD, Rep, and DinG further resolve R-loops by unwinding RNA–DNA hybrids and restoring duplex DNA (Merrikh et al., 2012; Urrutia-Irazabal et al., 2021; Bharati et al., 2022; Mugunthan et al., 2025). Topoisomerases contribute indirectly by modulating DNA supercoiling and relieving torsional stress generated during transcription. Genetic disruption of these enzymes results in elevated R-loop levels, replication stress, chromosomal fragmentation, and heightened sensitivity to DNA-damaging agents. Thus, R-loop homeostasis is a central component of bacterial genome maintenance systems (Raghunathan et al., 2018).

### 4.2 R-loops and stress-induced mutagenesis

Beyond their structural impact, R-loops are increasingly recognized as contributors to localized mutagenesis. The displaced ssDNA strand within an R-loop is chemically vulnerable and prone to cytosine deamination, oxidative base damage, and cleavage by structure-specific nucleases (Skourti-Stathaki et al., 2014; Chatzidoukaki et al., 2021; Jalan et al., 2024). Under antibiotic-induced oxidative stress, reactive oxygen species can further exacerbate damage within these exposed regions. When replication forks encounter R-loops, fork stalling and collapse may occur, triggering recombination-based repair or translesion synthesis pathways that are inherently error-prone (Chatzidoukaki et al., 2021; Jalan et al., 2024).

Importantly, R-loop–associated mutagenesis is not randomly distributed across the genome. R-loops form mainly at actively transcribed loci, so mutations often accumulate in highly expressed stress-responsive genes (Yan et al., 2019; Jiang et al., 2023). These genes frequently encode efflux pumps, metabolic regulators, stress-response proteins, or virulence factors. This links transcriptional activation, structural vulnerability, and mutagenic repair in a way that supports adaptive evolution under stress. Rather than increasing mutations across the whole genome, R-loops may concentrate genetic variation in regions most important for survival under selective pressure (Chen et al., 2018; Chacón et al., 2026).

Moreover, R-loops can promote larger-scale genome rearrangements. Persistent RNA–DNA hybrids may stimulate homologous recombination, deletions, duplications, or transposition events, contributing to genome plasticity. In the context of prolonged antibiotic exposure, such structural alterations can accelerate the acquisition or amplification of resistance determinants (Chatzidoukaki et al., 2021; Ortega et al., 2021; Merrikh et al., 2012; Urrutia-Irazabal et al., 2021). Thus, R-loops possibly function as structural catalysts that connect environmental stress with enhanced evolvability.

### 4.3 Cruciform and hairpin DNA: roles in genome stability and stress-induced regulation

Cruciform and hairpin DNA structures arise from inverted repeat sequences that extrude under negative supercoiling, a common feature of bacterial chromosomes and plasmids. These non-B DNA conformations form dynamically during transcription and replication and can act as topological sensors that link DNA superhelicity to gene regulation (Wu et al., 1988; Bacolla et al., 2016). Experimental studies have demonstrated direct regulatory roles for these structures. Horwitz (1989) showed that a cruciform-forming sequence inserted into the −35 region of an *Escherichia coli* promoter significantly altered transcription *in vitro*. In the supercoiling-sensitive *leu-500* promoter, Chen et al. (1992) demonstrated that transcription-induced negative supercoiling activates promoter function. Later on, an elegant study further reported sequence-dependent extrusion of a small hairpin at bacteriophage N4 promoters, indicating that promoter-associated hairpins can serve as regulatory recognition elements (Dai et al., 1998).

Cruciforms have also been visualized directly in plasmid DNA. Using atomic force microscopy, Shlyakhtenko et al. (1998) demonstrated that cruciforms form and remain highly dynamic in negatively supercoiled plasmids, while the same group later showed that reversible cruciform transitions can function as molecular switches that alter DNA architecture (Shlyakhtenko et al., 2000). Inverted repeats and repetitive DNA also contribute to genome plasticity and horizontal gene transfer. In *Helicobacter pylori*, repetitive DNA promotes recombination and chromosomal rearrangements that facilitate adaptation (Aras et al., 2003). Similar motifs are abundant in mobile genetic elements carrying antibiotic resistance genes, linking DNA secondary structures to genome remodeling and resistance dissemination (Khedkar et al., 2022). These elements are major targets of prokaryotic immune systems, highlighting their central role in microbial evolution (Mayo-Muñoz et al., 2023).

Although cruciform and hairpin DNA can regulate transcription and genome dynamics, they may also stall replication and trigger DNA breaks if not properly resolved. Overall, these structures function as stress-responsive elements that integrate DNA topology, gene expression, and genome plasticity in microbial adaptation.

### 4.4 Therapeutic implications of targeting R-loop homeostasis

Because R-loops regulate transcriptional stress, genome instability, and adaptive mutation, their regulatory pathways may offer promising antimicrobial targets. However, this area remains largely unexplored. Inhibiting RNase H or specific helicases could increase R-loop accumulation beyond tolerable levels, causing lethal genome instability or replication failure in antibiotic-stressed bacteria (Amon and Koshland, 2016; Lang et al., 2017). Alternatively, controlling R-loop resolution may reduce localized mutagenesis and limit resistance development during prolonged treatment as demonstrated in yeast (Amon and Koshland, 2016; Card et al., 2021).

Targeting R-loop metabolism poses challenges, particularly because RNA–DNA hybrid processing is conserved across organisms. However, bacterial-specific helicases, differences in RNase H isoforms, and the unique coupling of transcription and translation in prokaryotes offer potential avenues for selective intervention (O’Connell et al., 2013; Wollman et al., 2024). Combination strategies that pair R-loop–modulating agents with conventional antibiotics could exploit stress-induced vulnerabilities while limiting adaptive escape.

Overall, R-loops exemplify how transient nucleic acid structures can shape microbial genome dynamics. By linking transcriptional activity to structural instability and adaptive mutation, R-loops emerge not merely as byproducts of gene expression but as active drivers of genome plasticity and resistance evolution (Amon and Koshland, 2016; Lang et al., 2017; Card et al., 2021).

## 5 Triplex DNA: emerging roles and unresolved questions

Beyond G-quadruplexes, R-loops, and Z-DNA, triplex DNA (H-DNA) represents another non-canonical conformation with potential regulatory and mutagenic roles in microbial genomes. Although far less characterized in prokaryotes, triplex-forming sequences occur in repetitive and regulatory regions and may influence transcription, replication, and recombination under conditions of negative supercoiling.

### 5.1 Triplex DNA and H-DNA structures

Triplex DNA, often referred to as H-DNA when formed intramolecularly, arises when a third DNA strand binds within the major groove of duplex DNA through Hoogsteen or reverse Hoogsteen hydrogen bonds. This structure typically forms at homopurine–homopyrimidine mirror repeats and is favored under negative supercoiling and partial strand separation that are common during active transcription (Mirkin, 1999; Biet et al., 2003; Pasquier et al., 2017; Hwang et al., 2025).

Although triplex DNA has been extensively studied in eukaryotic systems, particularly in relation to genome instability and disease-associated repeat expansions, analogous triplex-forming sequences are present in bacterial genomes, especially within repetitive or regulatory regions (Walter et al., 2001; Holder et al., 2015; Hisey et al., 2024). In principle, triplex formation could interfere with transcriptional machinery, stall replication forks, or stimulate recombination events (Tiwari et al., 2016; Bacola et al., 2016; Matos-Rodrigues et al., 2023). Such effects might contribute to localized genome instability or provide an additional layer of transcriptional control during stress adaptation (Zhang et al., 2022; Roy et al., 2024).

However, experimental validation of triplex DNA function in prokaryotes remains sparse. The transient and context-dependent nature of triplex formation poses technical challenges for detection and functional characterization. Consequently, while computational predictions highlight structural potential, the physiological relevance of triplex DNA in bacteria remains largely unresolved (Amato et al., 2013; Conlon et al., 2016; Dey et al., 2021; Stojowska-Swędrzyńska et al., 2026). Addressing this gap will require integrated genomic, biochemical, and single-molecule approaches to determine whether triplex structures represent rare topological by-products or bonafide regulatory elements within microbial genomes.

## 6 Alternative nucleic acid structures in antibiotic persistence and tolerance

Antibiotic resistance is often framed as a consequence of stable genetic mutations; however, many treatment failures arise from phenotypic adaptations that do not involve permanent DNA sequence changes (Joshi et al., 2015; Card et al., 2021). Persistence and tolerance enable small bacterial subpopulations to survive transient or prolonged antibiotic exposure by entering dormant, slow-growing, or metabolically reprogrammed states. These phenotypes reduce antibiotic efficacy because most bactericidal agents target active cellular processes such as replication, transcription, or cell wall synthesis. Increasingly, alternative nucleic acid structures instead of only protein-centered regulatory pathways are being recognized as contributors to the transcriptional heterogeneity and regulatory plasticity that underlie these survival strategies (Lang et al., 2017; Shao et al., 2020; Quigley and Lewis, 2022; Kitzenberg et al., 2022; Stojowska-Swędrzyńska et al., 2026).

### 6.1 Structural modulation of transcriptional heterogeneity

Alternative DNA and RNA conformations, including G-quadruplexes and R-loops, can influence gene expression by altering promoter accessibility, transcription elongation dynamics, and RNA stability (Lang et al., 2017; Raghunathan et al., 2018; Niehrs and Luke, 2020; Xie and Dean, 2025). Because these structures form in a context-dependent and reversible manner, they introduce stochastic variability into transcriptional outputs across a genetically identical population. For example, a promoter capable of forming a G-quadruplex may fluctuate between transcriptionally permissive and repressive conformations depending on local supercoiling, ionic conditions, or metabolic state (Chaudhuri et al., 2024; Sultan et al., 2025). Similarly, transient R-loop formation at highly transcribed loci can slow or stall RNA polymerase, affecting transcript abundance and downstream regulatory cascades (Lang et al., 2017; Raghunathan et al., 2018; Briggs et al., 2018).

Such structural variability may contribute to bistable gene expression patterns, a hallmark of persister formation (Shan et al., 2017). Genes involved in toxin–antitoxin systems, stringent response pathways, and global stress regulators are often tightly controlled and sensitive to transcriptional perturbations (LeRoux et al., 2020). Even modest structural modulation at these loci could bias a subset of cells toward dormancy or growth arrest, generating phenotypic diversity within the population (Makarova et al., 2009; Wang and Wood, 2011). In this framework, alternative nucleic acid structures function as architectural switches that expand the range of transcriptional states available to bacterial cells.

### 6.2 Structural contributions to persistence and tolerance

Beyond generating heterogeneity, alternative structures may actively stabilize stress-adapted states. G-quadruplex formation within regulatory regions of stress-response genes could sustain altered transcriptional programs during prolonged antibiotic exposure (Hänsel-Hertsch et al., 2018). R-loops, through their effects on replication dynamics and localized genome instability, may further reinforce stress signaling pathways or trigger adaptive responses that enhance survival (Shankar et al., 2020). Importantly, these structural mechanisms operate independently of stable resistance mutations, allowing bacteria to endure antibiotic challenge while maintaining genetic flexibility (Wimberly et al., 2013; Card et al., 2021; Zheng et al., 2022; Sultan et al., 2025).

Over time, persistence and tolerance increase the probability that true resistance mutations will emerge, as surviving cells continue to replicate once antibiotic pressure subsides. By promoting transcriptional plasticity and stress resilience, alternative nucleic acid structures may therefore serve as indirect facilitators of resistance evolution (Levin-Reisman et al., 2017; Cotten and Davis, 2023). Understanding how these conformational states intersect with classical regulatory networks could reveal new strategies to disrupt persister formation and improve antibiotic efficacy.

## 7 Targeting non-canonical nucleic acid structures for antimicrobial drug development

Noncanonical nucleic acid structures represent an emerging antimicrobial target class that shifts intervention from protein-centered pathways toward genome architecture and regulatory topology. Whereas conventional antibiotics primarily inhibit essential proteins involved in cell wall synthesis, translation, DNA replication, or metabolism, targeting alternative DNA and RNA conformations such as G-quadruplexes and R-loops aims to interfere directly with transcriptional control, replication dynamics, and adaptive stress circuitry (Hänsel-Hertsch et al., 2018; Chaudhuri et al., 2024; Sultan et al., 2025). Because these mechanisms operate upstream of many classical resistance determinants, structural targeting may provide mechanistic orthogonality to existing antibiotics and reduce the likelihood of cross-resistance (Baedeker and Möller, 2026). Although no antimicrobial agents that directly target noncanonical nucleic acid structures have yet entered clinical trials, related therapeutic successes demonstrate that nucleic acid-directed strategies are clinically feasible. For example, the FDA-approved antisense drugs Eteplirsen and Nusinersen validate the ability of sequence-specific oligonucleotides to modulate RNA structure and function in patients, while riboswitch-targeting antibiotic programs such as Ribocil provide proof-of-concept that structured RNA elements can serve as tractable antibacterial targets (Mullard, 2023; Connelly et al., 2016).

Among alternative nucleic acid conformations, G-quadruplexes currently provide the strongest experimental basis for antimicrobial development. Small molecules that stabilize guanine tetrad structures have been shown in bacteria to suppress transcription of genes involved in virulence, metal homeostasis, and stress adaptation (Yuan et al., 2013). In multidrug-resistant *Klebsiella pneumoniae*, conserved promoter G-quadruplex motifs were experimentally validated in regulatory loci including *mgtA* and *entA*, and ligand-mediated stabilization reduced gene expression and impaired bacterial growth (Shankar et al., 2020; Chaudhuri et al., 2024). Similar observations in *Staphylococcus aureus* demonstrated that G-quadruplex-binding compounds inhibit growth and attenuate expression of genes linked to pathogenic fitness (Sultan et al., 2025).

These ligands generally act through π–π stacking interactions with exposed guanine tetrads, while additional groove and loop contacts contribute to selectivity (Yuan et al., 2013; Kaplan et al., 2016; Chaudhuri et al., 2024). Although many early G-quadruplex ligands were first developed in eukaryotic systems, their antimicrobial relevance depends on exploiting sequence and topological differences unique to microbial genomes. In bacteria, predicted G-quadruplex-forming sequences are enriched within virulence genes, transcriptional regulators, and metabolic control regions, suggesting that selective stabilization could repress adaptive pathways required during antibiotic stress (Rawal et al., 2006; Wells, 2009; Shao et al., 2020; Kumar et al., 2021). Conversely, inappropriate destabilization of endogenous structural equilibria may disrupt tightly coordinated transcriptional programs essential for survival.

Peptide nucleic acids (PNAs) and CRISPR-based antimicrobials offer complementary sequence-specific approaches to target structured genomic regions, but their clinical translation remains limited by delivery barriers. PNAs exhibit poor penetration across bacterial cell envelopes, particularly in Gram-negative species, and typically require conjugation to cell-penetrating peptides or other carrier systems to achieve intracellular activity (Sully et al., 2017; Moustafa et al., 2021). On the other hand, cationic phosphorodiamidate morpholino oligomers are reported to efficiently prevent growth of *Escherichia coli in vitro* and *in vivo* (Mellbye et al., 2010). Similarly, CRISPR-based antimicrobials depend on bacteriophages, conjugative plasmids, or nanoparticle platforms for delivery, each of which faces constraints related to host range, stability, and *in vivo* efficiency (Bikard and Barrangou, 2017; Tao et al., 2022). Despite these limitations, continued improvements in delivery technologies may enable programmable targeting of noncanonical nucleic acid structures as a clinically relevant antimicrobial strategy.

### 7.1 Strategic opportunities and selectivity considerations

From a drug development perspective, several strategic advantages emerge. First, targeting noncanonical nucleic acid structures may interfere with regulatory hubs rather than single enzymatic steps, potentially producing broader phenotypic consequences (Shankar et al., 2020; Chaudhuri et al., 2024; Sultan et al., 2025). For example, stabilizing a G-quadruplex within a promoter controlling efflux pumps or biofilm-associated genes could sensitize bacteria to conventional antibiotics. Similarly, modulating R-loop dynamics by promoting excessive accumulation to induce lethal genome instability or by suppressing stress-induced mutagenesis may offer a route to limit adaptive resistance evolution (Levin-Reisman et al., 2017).

Second, nucleic acid structure targeting may reduce the likelihood of rapid resistance development. Because structural motifs depend on sequence context and DNA topology rather than a single protein active site, mutational escape could carry substantial fitness costs. Moreover, combination therapies that pair structure-targeting agents with classical antibiotics could exploit stress-induced vulnerabilities while preventing adaptive mutation pathways.

However, achieving microbial selectivity is a central challenge. G-quadruplexes, R-loops, and other noncanonical structures are also present in host cells, raising concerns about cytotoxicity and off-target effects (Wimberly et al., 2013; Raghunathan et al., 2018). Rational design must therefore exploit differences in sequence composition, loop architecture, topology, and intracellular ionic environments between microbial and human nucleic acid structures. For example, microbial genomes often contain species-specific G-quadruplex motifs associated with virulence genes, resistance determinants, or essential regulatory loci that are absent from equivalent host pathways. In addition, bacterial-specific regulatory contexts, including transcription–translation coupling and distinct helicase repertoires, provide opportunities to selectively perturb R-loop metabolism without broadly affecting eukaryotic nucleic acid homeostasis (Huppert and Balasubramanian, 2007; Crossley et al., 2019).

Several complementary strategies may further mitigate off-target effects. Structure-guided medicinal chemistry can optimize ligands to recognize unique loop sequences or topological conformations enriched in microbial targets while reducing affinity for host structures. Conditional activation approaches, such as prodrugs that are unmasked by bacterial enzymes or reductive intracellular environments, can restrict activity to infected cells. Targeted delivery systems that include peptide-conjugated peptide nucleic acids (PNAs), bacteriophage-mediated vectors, and nanoparticle-based formulations can concentrate therapeutics within bacterial populations and minimize host exposure (Good and Nielsen, 1998; Nielsen, 2001; Geller et al., 2003). In parallel, transcriptomic and cytotoxicity profiling in mammalian cells should be incorporated early in development to identify compounds with narrow and selective activity windows.

Beyond small molecules, alternative modalities may enhance specificity. Antisense oligonucleotides, PNAs, or CRISPR-guided approaches could be engineered to induce or stabilize specific structural transitions within bacterial genomes (Good and Nielsen, 1998; Nielsen, 2001; Bikard et al., 2014; Cadoni et al., 2021). Advances in structural biology, single-molecule imaging, and genome-wide mapping technologies will be critical for identifying high-confidence targets, validating *in vivo* relevance, and confirming selective engagement of microbial rather than host nucleic acid structures.

Off-target effects remain a major challenge because G-quadruplexes, R-loops, and structured RNAs are also present in host cells. Therefore, therapeutic design must emphasize pathogen selectivity through species-specific structural motifs, sequence-targeted PNAs or antisense oligonucleotides, and targeted delivery systems such as peptides, phages, or nanoparticles. Conditional activation strategies and early cytotoxicity/off-target profiling will also be essential for safe clinical translation as also highlighted by many scientific contributions (Cadoni et al., 2021; Tao et al., 2022; Li et al., 2025).

Collectively, targeting noncanonical nucleic acid structures may emerge as a next big thing in antimicrobial drug discovery. By intervening at the level of genome structure and regulatory dynamics rather than protein catalysis alone, this strategy holds potential to complement existing therapies, suppress resistance emergence, and broaden the conceptual framework of antimicrobial intervention.

### 7.2 Potential for clinical translation and ongoing trials

Although no antimicrobial agents directly targeting noncanonical nucleic acid structures have yet progressed to infectious disease clinical trials, translational proof-of-concept has emerged from oncology. The G-quadruplex stabilizers CX-3543 (Quarfloxin) and CX-5461 (Pidnarulex) represent the first clinically evaluated G4-targeting compounds in humans (Drygin et al., 2008; Xu et al., 2017). CX-5461 has completed multiple Phase I clinical trials in patients with hematologic and solid malignancies and remains under active clinical investigation (NCT02719977; NCT04890613; NCT06606990) (Xu et al., 2017; Xu and Hurley, 2022; Li et al., 2025). These studies demonstrated that G-quadruplex structures are pharmacologically tractable targets *in vivo* and established the feasibility of therapeutically modulating noncanonical nucleic acid architectures. Although current trials focus on cancer, the same conceptual framework is increasingly being explored in microbial systems, where G-quadruplexes regulate virulence, stress adaptation, and antimicrobial resistance genes. Thus, existing clinical development of G4-targeting compounds provides an important translational foundation for future antimicrobial applications.

## 8 Challenges, methodological limitations, and future directions

Although interest in alternative nucleic acid structures has expanded considerably, defining their physiological roles in microbial systems remains technically and conceptually challenging. A central limitation lies in detection. Many experimental approaches rely on structure-stabilizing ligands, conformation-specific antibodies, or engineered probes that may alter the equilibrium of transient structures such as G-quadruplexes, R-loops, or Z-DNA. Chemical stabilization can artificially enhance structure persistence, raising concerns that observed phenotypes reflect experimental perturbation rather than native biology (Marsico et al., 2019; Chambers et al., 2015; Hänsel-Hertsch et al., 2018; Crossley et al., 2019). Similarly, antibody-based enrichment methods may exhibit cross-reactivity or bias toward highly stable conformations, potentially underrepresenting dynamic or short-lived intermediates. Recent primary studies using catalytically inactive RNase H1-based mapping and orthogonal chemical approaches have improved confidence in native R-loop detection and demonstrated that methodological choice can substantially influence biological interpretation (Yan et al., 2019; Wang et al., 2021; Miller et al., 2022; Wulfridge and Sarma, 2024; Li et al., 2025).

Another major challenge is distinguishing causality from correlation. Alternative nucleic acid structures frequently accumulate under stress conditions, such as oxidative damage, replication inhibition, or antibiotic exposure. This can make it difficult to determine whether they actively drive regulatory changes or arise as secondary consequences of perturbed cellular processes. For example, increased R-loop formation during antibiotic treatment may promote mutagenesis, but it may also reflect transcriptional dysregulation induced by drug stress. Addressing this issue requires targeted perturbation strategies, including synonymous sequence substitutions that disrupt a predicted motif without altering encoded proteins, inducible expression of structure-resolving helicases or RNase H enzymes, and use of ligands with matched inactive analogs as controls. These approaches enable direct comparison of strains that differ only in their capacity to form a given structure, thereby establishing whether the structure itself is responsible for changes in transcription, genome stability, or antibiotic susceptibility (Chambers et al., 2015; Krishnan et al., 2016; Martini et al., 2022).

Technical barriers also extend to resolution and quantification. Many genome-wide mapping strategies provide population-averaged signals that obscure cell-to-cell heterogeneity, an important factor in persistence and tolerance. Moreover, transient conformational transitions are difficult to capture using static sequencing-based methods. Single-molecule and real-time approaches can address this limitation by monitoring structure formation and dissolution during replication or transcription, while single-cell sequencing can identify whether alternative structures are enriched in rare persister subpopulations rather than across the entire culture (Noer et al., 2016; Cristini et al., 2018).

Future progress toward antimicrobial translation will depend on integrating these methodological advances with functional validation. High-resolution structure-specific sequencing can pinpoint candidate motifs associated with virulence genes, resistance determinants, or essential operons. Precise genetic disruption of these motifs can then test whether they are required for microbial fitness or drug tolerance. Single-molecule imaging can reveal when and under what environmental conditions structures form, helping to define the most vulnerable stages for therapeutic intervention. Chemical biology tools that selectively stabilize or destabilize individual motifs will further enable target engagement studies and structure–activity optimization. Coupling these approaches with transcriptomics, proteomics, and experimental evolution will allow researchers to determine whether pharmacological targeting of alternative nucleic acid structures imposes durable fitness costs or whether resistance readily emerges. Ultimately, a systems-level framework incorporating DNA topology, transcriptional regulation, and genome stability will be necessary to identify the most tractable structural targets and to evaluate their feasibility as next-generation antimicrobial strategies.

## 9 Conclusions

Noncanonical nucleic acid structures such as, G-quadruplexes, R-loops, Z-DNA, and triplex DNA are not byproducts but active regulators of microbial genome function. By reshaping promoter accessibility, transcriptional dynamics, and genome stability, they drive phenotypic heterogeneity, persistence, and the emergence of antibiotic resistance (Palmer and Kishony, 2013; Wimberly et al., 2013; Levin-Reisman et al., 2017; Shankar et al., 2020). These structures couple environmental stress, including antibiotic exposure, to genome plasticity, providing a direct mechanistic link between transcription and adaptive mutagenesis (Lang et al., 2017; Card et al., 2021). This perspective reframes resistance as an emergent property of genome architecture rather than solely protein-level changes or gene acquisition. Targeting DNA topology and RNA–DNA hybrid metabolism therefore represents a promising therapeutic axis to suppress adaptive evolution and enhance antibiotic efficacy (Palmer and Kishony, 2013). This warrants a deeper integration of structural nucleic acid biology with systems and chemical approaches.
